# Combined Fluoxetine and Metformin Treatment Potentiates Antidepressant Efficacy Increasing IGF2 Expression in the Dorsal Hippocampus

**DOI:** 10.1155/2019/4651031

**Published:** 2019-01-21

**Authors:** Silvia Poggini, Maria Teresa Golia, Silvia Alboni, Giampaolo Milior, Livio Pepè Sciarria, Aurelia Viglione, Gloria Matte Bon, Nicoletta Brunello, Stefano Puglisi-Allegra, Cristina Limatola, Laura Maggi, Igor Branchi

**Affiliations:** ^1^Center for Behavioral Sciences and Mental Health, Istituto Superiore di Sanità, Rome, Italy; ^2^PhD Program in Behavioral Neuroscience, Sapienza University of Rome, Rome, Italy; ^3^Department of Physiology and Pharmacology, Laboratory Affiliated to Istituto Pasteur Italia, Sapienza University of Rome, Rome, Italy; ^4^Department of Life Sciences, University of Modena and Reggio Emilia, Modena, Italy; ^5^Inserm U1127, CNRS UMR7225, Sorbonne Universités, UPMC Univ Paris 6 UMR S1127, Institut du Cerveau et de la Moelle épinière, Paris 75013, France; ^6^PhD Program in Neuroscience, Scuola Normale Superiore di Pisa, Pisa, Italy; ^7^Department of Psychology, Sapienza University of Rome, Rome, Italy; ^8^Santa Lucia Foundation (IRCCS Fondazione Santa Lucia), Rome, Italy; ^9^IRCCS Neuromed, Pozzilli, Isernia, Italy

## Abstract

An increasing number of studies show that selective serotonin reuptake inhibitors (SSRIs) exert their therapeutic action, at least in part, by amplifying the influence of the living environment on mood. As a consequence, when administered in a favorable environment, SSRIs lead to a reduction of symptoms, but in stressful conditions, they show limited efficacy. Therefore, novel therapeutic approaches able to neutralize the influence of the stressful environment on treatment are needed. The aim of our study was to test whether, in a mouse model of depression, the combined administration of SSRI fluoxetine and metformin, a drug able to improve the metabolic profile, counteracts the limited efficacy of fluoxetine alone when administered in stressful conditions. Indeed, metabolic alterations are associated to both the onset of major depression and the antidepressant efficacy. To this goal, adult C57BL/6 male mice were exposed to stress for 6 weeks; the first two weeks was aimed at generating a mouse model of depression. During the remaining 4 weeks, mice received one of the following treatments: vehicle, fluoxetine, metformin, or a combination of fluoxetine and metformin. We measured liking- and wanting-type anhedonia as behavioral phenotypes of depression and assessed the expression levels of selected genes involved in major depressive disorder and antidepressant response in the dorsal and ventral hippocampus, which are differently involved in the depressive symptomatology. The combined treatment was more effective than fluoxetine alone in ameliorating the depressive phenotype after one week of treatment. This was associated to an increase in IGF2 mRNA expression and enhanced long-term potentiation, specifically in the dorsal hippocampus, at the end of treatment. Overall, the present results show that, when administered in stressful conditions, the combined fluoxetine and metformin treatment may represent a more effective approach than fluoxetine alone in a short term. Finally, our findings highlight the relevance of polypharmacological strategy as effective interventions to increase the efficacy of the antidepressant drugs currently available.

## 1. Introduction

Selective serotonin reuptake inhibitors (SSRIs) are the most commonly prescribed drugs for the treatment of major depressive disorder (MDD), which constitutes an enormous medical, individual, societal, and economical challenge and afflicts up to 10–15% of the population worldwide [1]. However, the efficacy of SSRIs is variable and incomplete: 60–70% of patients do not experience remission and 30–40% do not show a significant response [2]. To explain such incomplete efficacy, a novel hypothesis—named *undirected susceptibility to change*—posits that the increase in serotonin levels induced by SSRIs does not affect mood *per se* but enhances brain plasticity and thus amplifies the influence of the environment on the individual [3–6]. Therefore, SSRI treatment has not a univocal effect, but in a favorable environment, it would lead to a reduction of symptoms while in a stressful environment, it has limited efficacy and may even lead to a worse prognosis [7].

A number of evidence support a role for serotonin in increasing brain plasticity and enhancing susceptibility to the environment [3, 8, 9]. In addition, the *undirected susceptibility to change* hypothesis has been recently demonstrated at preclinical and clinical levels. In preclinical studies, it has been shown that fluoxetine (FLX), one of the most commonly prescribed SSRIs, affects the molecular and behavioral depression-like phenotype according to the quality of the living environment: when administered in an enriched environment, it led to an improvement while, when administered in a stressful environment, FLX treatment led to a worsening of depression-like endpoints such as an enhanced anhedonic behavior and a reduced neurogenesis [5, 10]. At a clinical level, it has been demonstrated that the commonly used SSRI citalopram amplifies the influence of the living conditions on mood, confirming that SSRI effects are affected by the environment [11].

The SSRI action on brain plasticity and susceptibility to the environment opens new perspectives on how to improve the efficacy of these antidepressants by improving the quality of the patients' living environment. However, often, it is not possible to act on the environment because of constraints due to patient's personal history and unchangeable life circumstances. In these cases, the pharmacological modulation of the factors underlying the link between the living environment and SSRI action represents a novel and desirable strategy to improve treatment outcome in patients living in adverse conditions, very common in depressed patients.

Metabolism is markedly affected by the quality of the living environment. For instance, having an active physical and social life profoundly modulates metabolic markers [12, 13]. In turn, the metabolic profile affects vulnerability to MDD and antidepressant efficacy. As an example, normalization of metabolic markers has been associated to remission following antidepressant treatment, while no change occurs in nonremitters [14]. Therefore, modification of metabolism represents a potential approach to modulate the interplay between the environment and SSRIs in order to improve treatment outcome.

The aim of the present study was to assess whether the pharmacological modulation of metabolism may improve the limited efficacy of FLX when administered in a stressful environment. To this purpose, we used metformin (MET), a widely used drug to treat type II diabetes and other metabolic syndromes [15]. It affects the metabolic profile at both peripheral and central levels since it crosses the blood-brain barrier [16, 17]. Though the underlying molecular mechanisms of MET action are yet to be fully determined, it has been reported in preclinical models that MET affects brain plasticity, increasing long-term potentiation (LTP) in the hippocampal CA1 region [18] and modulating neurotrophic factor levels, such as the brain-derived neurotrophic factor (BDNF) [19].

Our prediction was that the combined FLX and MET treatment is more effective than FLX alone in counteracting a depression-like phenotype in a stressful environment. According to our hypothesis, such enhanced efficacy is ascribable to the combined action of the two drugs: FLX increases brain plasticity, favoring a change in mood, while MET, which affects the metabolic profile, drives the change toward an improvement. To this aim, we measured BDNF expression and LTP as molecular and cellular markers of neural plasticity, in addition to liking- and wanting-type anhedonia as endpoints of depression-like response.

## 2. Materials and Methods

### 2.1. Animals

C57BL/6 male mice 12–15 weeks old were used and kept under a 12-hour light-dark cycle at 22–25°C. Mice were purchased at Envigo Italia (Udine, Italy). All procedures were carried out in accordance with the European law (EEC Council Directive 2010/63/UE86/609 1987), Italian legislation on animal experimentation (Decreto Legislativo 26/2014). In addition, animals were routinely examined for signs of discomfort as indicated by the animal care and use of the National Academy of Sciences of USA guidelines (National Research Council 2003).

### 2.2. Housing Condition

For the entire duration of the experiment, animals were housed in the IntelliCage system (TSE Systems, NewBehavior AG, Zürich, Switzerland), which is an apparatus for automatic monitoring of mouse behavior. It consists of a large acrylic cage (20.5 cm high, 58 cm × 40 cm at the top, and 55 cm × 37.5 cm at the base, Model 2000 Tecniplast, Buguggiate, VA, Italy) with 4 walls separating each corner from the center so that they form 4 identical triangular conditioning chambers (15 × 15 × 21 cm). Animals have access to the chamber by entering a front hole (chamber entrance). Only one mouse at a time can enter the chamber. Once entered, it is identified through a transponder antenna system. The system is able to collect data about the number and duration of visits and the number, duration, and side (right or left) of nosepokes and licks. The floor of the cage was covered with bedding and contains four sleeping shelters in the center while on the top, a food rack is present filled with standard mouse chow (food ad libitum). An additional cage (SocialBoxes) was used to expand the existing IntelliCage to a multiarea system; thus, we increased the capacity of the system to test simultaneously more mice. One week before being moved to the IntelliCage, each animal was injected with a subcutaneous transponder (T-IS 8010 FDX-B; Datamars SA, Switzerland). Mice have been gradually habituated to the IntelliCage environment during a 14-day period (habituation period).

### 2.3. Treatment

After the first two weeks of stressful condition, aimed at inducing a depression-like phenotype, mice continued to be exposed to the unpredictable chronic mild stress (see below) for 4 weeks receiving one of the following treatments: VEH, FLX, MET, or FLX and MET together.

FLX (Fluoxetine HCl, Santa Cruz, USA) and MET (Metformin, Sigma-Aldrich, St Louis, MO, USA) were dissolved in water and in saccharin solution and delivered ad libitum in the drinking bottles for 4 weeks. Compared to injection, this administration method allows avoiding the stress due to the manipulation. The solutions were prepared according to the mouse average weight and daily water consumption in order to provide an average daily intake of 30 mg/kg of FLX [20] and 200 mg/kg of MET, respectively [21, 22]. Bottles with FLX and with FLX and MET were wrapped in tin foil to protect the substance from light. Metformin, fluoxetine, and their combination were dissolved in both water and saccharin solution to avoid that the saccharin preference could affect the amount of drug received. The average amount of fluoxetine or metformin administered did not differ among the experimental groups receiving the same compound. Though we did not perform a pharmacokinetic analysis, to our knowledge, no interaction between fluoxetine and metformin has been reported.

### 2.4. Environmental Conditions

All mice were exposed to the stressful condition for two weeks to induce the depression-like behavior. For the following 4 weeks, the subjects went on being exposed to the stressful condition or were exposed to the standard condition.

#### 2.4.1. Stressful Condition

Mice were exposed to unpredictable chronic mild stress procedure to induce depression-like behavior (Figure S1). To prevent habituation to stress, mice were exposed each day to a different stressful procedure, randomly chosen among the procedures provided by the IntelliCage. The procedures were *short open door*: the door to access water or saccharin solution remains open for only 1.5 seconds; *delay*: the door opens with a delay of 1, 1.5, 2, and 2.5 seconds after the first nosepoke; *open door 25%*: the door opens only following 25% of nosepokes; and a*ir puff*: when the mouse performs a visit, it has a 20% chance to receive an air puff (2 bar) which lasts 1 sec or until the animal leaves the corner. In the latter case, the doors remain closed. Once each one of these procedures ended, in order to reopen the doors and drink again, the animals had to leave the corner and start a new visit. The duration of each stressful procedure was randomly chosen: 12, 18, or 24 hrs. In addition, during the stressful condition, no shelter or tissue paper was provided.

#### 2.4.2. Standard Condition

Mice were socially housed in the IntelliCages and exposed to Plexiglas shelters of different colors and shapes (four red transparent Tecniplast plastic nest boxes and four white opaque boxes) and to tissue paper. New paper was provided every 5 days, and the plastic shelters were cleaned every week (Figure S2(a)).

### 2.5. Behavioral Tests

Behavioral endpoints investigated are liking- and wanting-type anhedonia. These were automatically assessed by the IntelliCage avoiding any bias or stress due to the experimenter.

#### 2.5.1. Liking-Type Anhedonia: Saccharin Preference

To assess liking-type anhedonia, we measured the saccharin preference. Two bottles were present in each corner of the IntelliCage, one containing tap water and the other containing the 0.1% saccharin solution; both were freely available 24/24 h. Water and saccharin solution were substituted every day. The position of water and saccharin in each corner was counterbalanced across the four corners. The saccharin preference was determined as follows: (saccharin solution consumed/saccharin solution consumed + water consumed) × 100. We measured the baseline saccharin preference across a two-day period (i) at the end of the habituation period, (ii) at the end of the first two weeks of exposure to the stressful condition (aimed at inducing the depression-like phenotype), (iii) 1 week after the beginning of the treatment period, and (iv) at the end of the treatment period. Mice were exposed to the saccharin solution only (all bottles filled with saccharin solution) during the first two days of the IntelliCage habituation period in order to make them used to the saccharin flavor. In the remaining 12 days, mice could choose between water and saccharin solution.

#### 2.5.2. Wanting-Type Anhedonia: Progressive Ratio Schedule

To assess wanting-type anhedonia, i.e., the drive for obtaining a reward, we used the progressive ratio reinforcement schedule that utilizes a multiplicative increase in the number of responses (nosepokes) required to dispense a unit of reinforce (i.e. access to saccharin). In particular, water was always accessible after one nosepoke while saccharin solution was accessible only after a specific number of nosepokes that increases progressively. After each series of 8 visits, the number of nosepokes required to access saccharin increases according to the following schedule: 1, 2, 3, 4, 5, 6, 7, 8, 10, 12, 16, 20, and 24. After reaching the 24 nosepoke level, mice had free access to saccharin following one nosepoke. The time for performing the nosepokes increased gradually according to the number of nosepokes requested from one to 24 sec. Mice were exposed to this test at the end of the habituation period, immediately before the treatment period and after both 1 and 4 weeks of treatment. To make the mice aware of the progressive ratio testing, the three LEDs on the top of each door were kept turned on throughout the test. Each test session lasted two days.

### 2.6. RNA Extraction and RT-RTqPCR

Following 4 weeks of treatment in the stressful condition, animals were sacrificed by decapitation, the brains were removed, and the dorsal and ventral parts of the hippocampus were dissected, rapidly frozen, and then stored at −80°C for further molecular analyses. The same animals tested for behavior were analyzed for mRNA expression. Total RNA, from the ventral and dorsal hippocampi, was prepared combining extraction with TRI Reagent® and GenElute™ Mammalian Total RNA Miniprep Kit and (Sigma Aldrich ®, Milan, Italy) as previously described [4]. Two *μ*g of total RNA was reverse transcribed using High-Capacity cDNA Reverse Transcription Kit (Thermo Fisher Scientific, Waltham, MA USA) in a final reaction volume of 20 *μ*L [23]. The cDNA was stored at −20°C until real-time PCR that was performed in Roche LightCycler® 480 (Roche Diagnostics GmbH, Roche Applied Science, Mannheim, Germany) using Power SYBR Green Mix (Life Technologies Corporation, Carlsbad, CA, USA). The following forward and reverse sequences were used at the final concentration of 150 nM: for IGF1 F5′-TGCTCTTCAGTTCGTGTG-3′ and R5′-ACATCTCCAGTCTCCTCAG-3′; for IGF2 F5′-CGCTTCAGTTTGTCTGTTCG-3′ and R5′-GGAAGTACGGCCTGAGAGGTA-3′; for BDNF F5′-CCATAAGGACGCGGACTTGTAC-3′ and R5′-AGACATGTTTGCGGCATCCAGG-3′; for p11 (S100a10) F5′-CTTCAAAATGCCATC CCAAA-3′ and R5′-TATTTTGTCCACAGCCAGAGG-3′, for leptin F5′-AAGAAGATCCCAGGGAGGA and R5′-TGATGAGGGTTTTGGTGTCA, and for glyceraldehydes-3-phosphate dehydrogenase (GAPDH) F5′-TTCGCAAAACAAGTTCACCA-3′ and 5′-TCGTTGTGGTTGTAAATGGAA-3′ as a house-keeping gene. Melt curve analyses and agarose gel separations were performed at the end of every RTqPCR to confirm formation of a single PCR product. The Ct (cycle threshold) value was determined by the LightCycler® 480 Software (Roche Diagnostics GmbH, Roche Applied Science, Mannheim, Germany), and mRNA expression was calculated with the ΔΔCt method with GAPDH as endogenous control as previously described [24]. Relative expression of the genes of interest was performed by using as calibrator (RQ value = 1) expression levels in the ventral hippocampi of vehicle-treated animals. All qPCR reactions based upon the same primer set were run in the same amplification plate to compare the levels of mRNA expression between the two parts of the hippocampus.

### 2.7. Electrophysiology

#### 2.7.1. Hippocampal Slice Preparation

In order to perform electrophysiological experiments, acute hippocampal slices were collected. At the end of the treatment period in the stressful condition, animals were anesthetized by inhalation of halothane (Sigma-Aldrich S.r.l., Milan, Italy) and decapitated. The brain was rapidly removed from the skull and immersed in ice-cold artificial cerebrospinal fluid (ACSF) solution composed of the following (in mM): NaCl 125, KCl 4, CaCl2 2.5, MgSO4 1.5, NaHPO4 1, NaHCO3 26, and glucose 10. ACSF was continuously bubbled with 95% O2 + 5% CO2 to maintain a pH close to 7.4.

Following removal, the brain was hemisected along the longitudinal fissure to separate the two hemispheres. Brain dissection was carried out according to the slicing plane chosen and the structure to be investigated. Specifically, for experiments on the ventral hippocampus, slices were cut perpendicular to the longitudinal axis from the temporal pole of the brain. For experiments on the dorsal hippocampus, coronal slices were cut from the frontal pole. Dorsal and ventral slices have been identified as the distance, in *μ*m, from the frontal and temporal pole, respectively (approximately from 400 to 1750 *μ*m). The brain tissues were blocked on the stage of a vibrating microtome (Thermo Scientific, USA), and 350 *μ*m-thick slices were cut in ice-cold ACSF. The slices were then transferred to an incubation chamber containing oxygenated ACSF, where they were allowed to recover for 1 h at 30°C prior to electrophysiological recording. After this period, the slices were transferred to the interface slice-recording chamber (BSC1, Scientific System Design Inc.) to perform experiments within 1–6 h after slice preparation. Dorsal and ventral slices were prepared from separate hemispheres of the same brain and were obtained alternately from the right or left hemisphere.

#### 2.7.2. Extracellular Field Recordings

For field recordings, individual slices were maintained at 30–32°C and superfused with ACSF at 2 mL/min by a peristaltic pump. A concentric bipolar stimulating electrode (SNE 100 × 50 mm long, Elektronik Harvard Apparatus GmbH) was placed in the stratum radiatum to stimulate Schaffer collateral fibers. Stimuli consisted of 100 *μ*s constant current pulses of variable intensities, applied at 0.05 Hz. A glass micropipette (0.5–1 M*Ω*) filled with ACSF was placed in the CA1 hippocampal region, at 200–600 *μ*m from the stimulating electrode, in order to measure orthodromically evoked field extracellular postsynaptic potentials (fEPSP). Stimulus intensity was adjusted to evoke fEPSP of amplitude about 50% of the maximal amplitude with minimal contamination by a population spike. Evoked responses were monitored online, and stable baseline responses were recorded for at least 10 min. Only the slices that showed stable fEPSP amplitudes were included in the experiments. LTP was induced by high-frequency stimulation (HFS) (1 train of stimuli at 100 Hz of 1 s duration), repeated after 30 min. To analyze the time course of the fEPSP slope, the recorded fEPSP was routinely averaged over 1 min (*n* = 3). The fEPSP slope changes following the LTP induction protocol at 31 and 61 min post tetanus were calculated with respect to those of the baseline (1 minute before induction). *N*/*n* refers to the number of slices on the total number of mice analyzed.

The paired-pulse ratio (PPR) was measured from responses to two synaptic stimuli at 50 ms interstimulus interval. PPR was calculated as the ratio between the fEPSP amplitude evoked by the second stimulus (A2) and that by the first (A1; A2/A1).

fEPSP were recorded and filtered (low pass at 1 kHz) with an Axopatch 200A amplifier (Axon Instruments, CA) and digitized at 10 kHz with an A/D converter (Digidata 1322A, Axon Instruments). Data acquisition was stored on a computer using pClamp 9 software (Axon Instruments) and analyzed offline with Clampfit 10 program (Axon Instruments).

### 2.8. Data and Statistical Analysis

All data were analyzed with one-way ANOVA with the statistical software StatView II (Abacus Concepts, CA, USA), comparing VEH versus FLX-, MET-, and FLX plus MET-treated mice. When a significant main effect was found, selected pairwise comparisons were made using Tukey's post hoc analysis.

## 3. Results

### 3.1. FLX and MET Combination Is Effective in Alleviating Depression-Like Behavior

As behavioral phenotypes of depression, we assessed the liking- and wanting-type anhedonia, which have been previously shown to be susceptible to stress and SSRI treatment [5, 10, 25].

**Figure 1:**
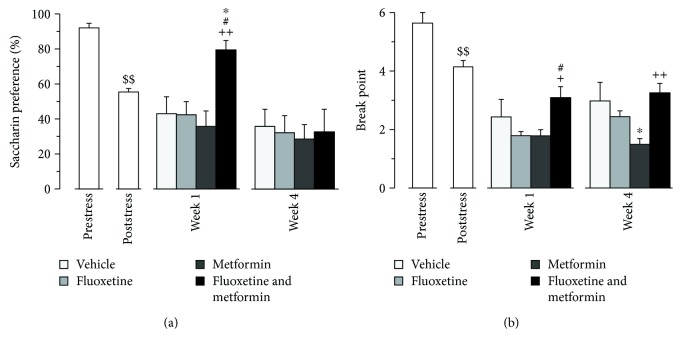
Effects of treatment with fluoxetine, metformin, or their combination on depression-like behavior. (a) Liking-type anhedonia. Saccharin preference significantly decreased following exposure to stressful procedure. After the first week of treatment, FLX-MET mice displayed an increased saccharin preference compared to both VEH, FLX, and MET mice. (b) Wanting-type anhedonia. The breakpoint level was significantly reduced after the unpredictable chronic mild stress. Following the first week of treatment, FLX-MET mice showed a significant increase of the breakpoint level compared to both FLX and MET mice. Treatments as indicated in the legend, *n* = 9 − 10 mice per group. $$*p* < 0.0001 pre- vs poststress, ^∗^*p* < 0.05 and ^∗∗^*p* < 0.001 vs VEH, ^#^*p* < 0.05 and ^##^*p* < 0.0001 vs FLX, and ^+^*p* < 0.05 and ^++^*p* < 0.001 vs MET. Data are presented as mean + SEM.

The two weeks of chronic stress before treatment was effective in inducing a depression-like profile. In particular, the saccharin preference (liking-type anhedonia) dropped from around 90 to 55 percent (*F*(1, 37) = 87.870, *p* < 0.0001, Figure 1(a)) and the breakpoint level (wanting-type anhedonia) was significantly reduced (*F*(1, 39) = 17.874, *p* < 0.0001, Figure 1(b)). No difference in weight between treated and control groups was found (data not shown), indicating that the stress procedure did not differentially affect the experimental groups. Following the induction of a depression-like profile, mice receiving the combination of FLX and MET showed an improvement of their behavioral phenotype when compared to those of the other experimental groups. Specifically, following 1 week of treatment, liking-type anhedonia was significantly affected by treatment (*F*(3, 34) = 6.126, *p* = 0.0019); post hoc analysis revealed that FLX-MET mice displayed an increased saccharin preference compared to VEH, FLX, and MET mice (*p* < 0.05, *p* < 0.05, and *p* < 0.001, respectively; Figure 1(a)). Wanting-type anhedonia was significantly affected by treatment as well (*F*(3, 35) = 3.047, *p* = 0.0414). FLX-MET mice showed a significant increase of the breakpoint level compared to both FLX and MET mice (*p* < 0.05 and *p* < 0.05, respectively; Figure 1(b)). At the end of the treatment, the prolonged exposure to stress (6 weeks) led to a marked anhedonic profile in all groups, flattening the potential differences in liking-type anhedonia. As for wanting-type anhedonia, a significant main effect of treatment was found (*F*(3, 35) = 4.329, *p* = 0.0107). In particular, FLX-MET mice reached a higher breakpoint level compared to MET mice (*p* < 0.001, Figure 1(b)).

All mice to be treated in the standard condition showed a significant increase of the depression-like phenotype following the two weeks of exposure to the stressful condition (Figures S2(b) and (c)). In particular, both liking-type anhedonia (*F*(1, 41) = 43.721, *p* < 0.0001) and wanting-type anhedonia (*F*(1, 40) = 10.681, *p* = 0.0022) were significantly reduced. Afterwards, when receiving VEH, FLX, MET, or FLX-MET in a standard condition, they showed no difference in depression-like behavior. In particular, all experimental groups showed a full recovery, displaying no anhedonic response, both at 1 and 4 weeks of treatment.

### 3.2. IGF2 mRNA Levels Are Increased in the Dorsal Hippocampus of Mice Receiving the Combined Treatment

To explore the molecular bases of treatment effect, we analyzed gene expression of selected targets reported to be involved in MDD and metabolism. In particular, we focused on IGF2 and IGF1, p11, BDNF, and leptin mRNA expression in the dorsal and ventral hippocampus. These hippocampal areas have been reported to be differently involved in MDD and antidepressant efficacy [26–30].

**Figure 2:**
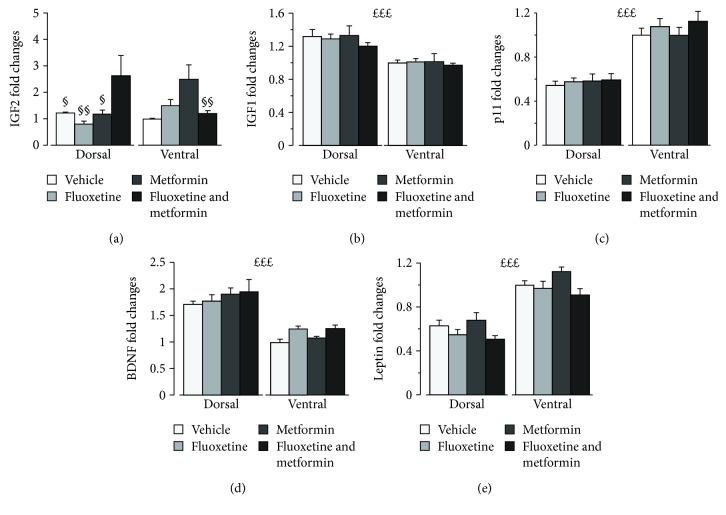
Effects of fluoxetine, metformin, or their combination on expression of genes involved in brain plasticity in the dorsal and ventral hippocampi. (a) IGF2 is significantly increased by the combined treatment compared to VEH and FLX alone, suggesting that this growth factor is involved in the antidepressant action of the FLX-MET treatment. Such effect concerned the dorsal hippocampus, where FLX-MET treatment increased IGF2 expression compared to all the other groups. (b) IGF1, (c) p11, (d) BDNF, and (e) leptin expression was not affected by treatment, but IGF1 and BDNF levels were overall higher in the dorsal hippocampus, while p11 and leptin were higher in the ventral hippocampus. Treatments as indicated in the legend, *n* = 6–8 mice per group. ^*£££*^*p* < 0.001, the main effect of the hippocampal region; ^§^*p* < 0.05 and ^§§^*p* < 0.01 vs FLX-MET in the dorsal region. Data are presented as mean + SEM.

IGF2 analysis revealed a significant main effect of treatment (*F*(3, 50) = 3.370, *p* = 0.0256) and a significant interaction treatment × hippocampal region (*F*(3, 50) = 5.912, *p* = 0.0015; Figure 2(a)). Post hoc analysis revealed that, overall, mice receiving the combined treatment showed higher IGF2 expression compared to those of the VEH and FLX groups. With regard to the dorsal hippocampus, they displayed higher IGF2 levels compared to VEH (*p* < 0.05), MET (*p* < 0.05), and FLX (*p* < 0.01). FLX-MET mice showed also significantly higher IGF2 levels in the dorsal region compared to the ventral region (*p* < 0.01). As for the other genetic markers investigated, IGF1, BDNF, p11, and leptin, we found no effect of treatment but a significant main effect of the hippocampal region (*F*s(1, 50) = 29.161, 96.221, 114.972, 126.865, *p*s < 0.001, Figures 2(b)–2(e)). In particular, IGF1 and BDNF levels were higher in the dorsal hippocampus, while p11 and leptin were higher in the ventral hippocampus.

### 3.3. LTP in the Dorsal and Ventral Hippocampal Regions Is Differentially Affected by Treatment

**Figure 3:**
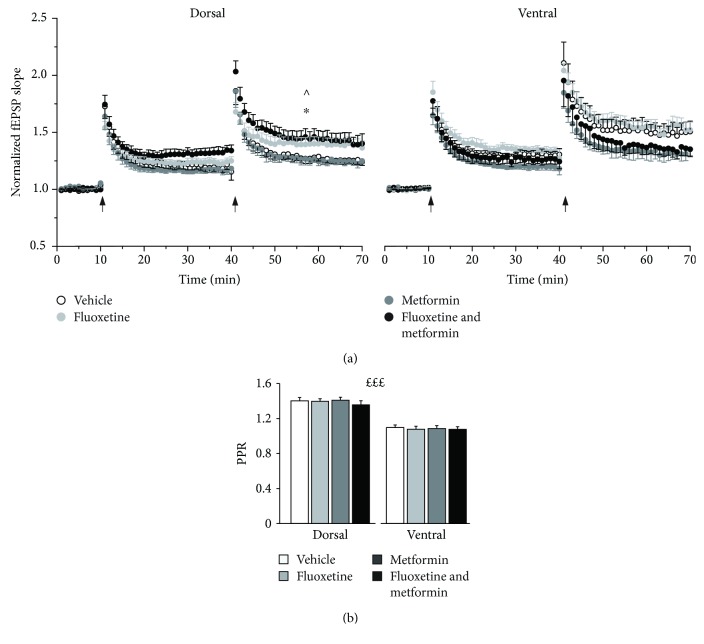
LTP in the dorsal and ventral hippocampus is differentially affected by fluoxetine, metformin, or their combination. (a) LTP from extracellular records in the dorsal and ventral hippocampi. The time course of fEPSP slope responses evoked at 0.05 Hz and normalized as detailed in Materials and Methods. Arrows indicate repeated spaced HFS (100 Hz trains of 1 sec duration, 30 minutes apart). Treatments as indicated in the legend (in the dorsal hippocampus, VEH: *n* = 12/8, FLX: *n* = 10/9, MET: *n* = 12/9, and FLX-MET: *n* = 10/7 and in the ventral hippocampus, VEH: *n* = 10/10, FLX: *n* = 8/8, MET: *n* = 9/8, and FLX-MET: *n* = 11/9). Note that in the dorsal hippocampus, FLX-MET mice show an increased LTP compared to MET or VEH mice and that in VEH, LTP is higher in the ventral compared to the dorsal hippocampus. Tukey's *t*-test post hoc analysis, 20 minutes after the second HFS. ^∗^*p* < 0.05 FLX-MET vs VEH; ^∧^*p* < 0.05 FLX-MET vs MET. (b) PPR. Bar histogram indicates averaged PPR values for the dorsal hippocampus (VEH: *n* = 20/12, FLX: *n* = 20/12, MET: *n* = 19/11, and FLX-MET: *n* = 21/12) and ventral hippocampus (VEH: *n* = 14/11, FLX: *n* = 16/11, MET: *n* = 21/12, and FLX-MET: *n* = 16/10). Note that PPR is not affected by treatments and it is higher in the dorsal compared to the ventral hippocampus. Treatments as indicated in the legend, ^*£££*^*p* < 0.001, the main effect of the hippocampal region. Data are presented as mean + SEM.

We explored plasticity processes in the CA1 hippocampal region by recording LTP evoked by two spaced (30 minutes apart) Schaffer collateral stimulations in both the dorsal and ventral hippocampus. Interestingly, during the second stimulation, the main effect of treatment emerged (*F*(3, 60) = 3.321, *p* = 0.026). In addition, the main effect on the hippocampal area and treatment × hippocampal region interaction were very close to reach statistical significance (*F*(1, 60) = 3.473, *p* = 0.067 and *F*(3, 60) = 2.523, *p* = 0.066, respectively). Post hoc analysis revealed that, in the dorsal hippocampus, FLX-MET-treated mice show an increased LTP amplitude (1.495 ± 0.065) compared to MET- (1.226 ± 0.061, *p* < 0.05) and VEH- (1.260 ± 0.058, *p* < 0.05, Figure 3(a), left) but not to FLX- (1.406 ± 0.069) treated mice. By contrast, in the ventral region, the combined treatment showed a trend toward a reduction of LTP amplitude compared to FLX and VEH alone (1.387 ± 0.058 vs 1.525 ± 0.065 and 1.479 ± 0.061, Figure 3(a), right) but was similar to MET alone (1.323 ± 0.0759). Finally, in the VEH group, the magnitude of LTP was higher in the ventral compared the dorsal hippocampus (*p* = 0.012).

With regard to PPR, a main effect on the hippocampal region was observed following treatment (*F*(1,139) = 157.357, *p* < 0.001), being its value higher on the dorsal hippocampus for all treatments (*p* < 0.001, Figure 3(b)). In the VEH group, PPR was 1.402 ± 0.034 and 1.081 ± 0.041, in the dorsal and ventral hippocampi, respectively. No treatment effect was observed.

## 4. Discussion

The present results show that the combination of FLX and MET administered in the stressful condition ameliorates the depression-like phenotype compared to FLX and VEH alone after one week but not after four weeks of treatment. The combination of FLX and MET led also to increased IGF2 expression and enhanced LTP, specifically in the dorsal hippocampus, at the end of treatment.

Previous findings by us [5, 10] indicate that FLX alone administered in a chronic stress condition has limited beneficial effects or leads to a worsening of depression-like behavior. This is in line with previous studies [31–34]. However, other studies found that mice treated with SSRI in a stressful environment show an improvement of the depression-like profile [35, 36]. Here, we confirm that, compared to VEH, FLX has limited beneficial effects when administered in adverse conditions. However, the cotreatment of MET and FLX counteracts the detrimental effects induced by the exposure to stress following 1 week of treatment. In particular, FLX and MET combination increases the saccharin preference to the level that the mice had before chronic stress compared to both VEH and FLX alone. Similar results have been found for wanting-type anhedonia, mice treated with the combined treatment showing a higher motivation to obtain the reward compared to those receiving FLX only. The results collected at four weeks show no difference among the experimental groups, indicating that the combined treatment has not long-lasting beneficial effects on depression-like behavior and should be used for subacute interventions. It is worth noting that, when administered in standard condition, treatments did not produce different effects, all experimental groups showing a recovery of the anhedonic profile at both weeks 1 and 4 (Figure S2). These results confirm that the MET-FLX combination is an effective therapeutic approach when administered to subjects living in stressful conditions and support our hypothesis that FLX treatment outcome depends on the quality of the environment [7].

To explore the molecular mechanisms associated to the therapeutic action of the combined treatment, we analyzed the expression of selected genes reportedly involved in MDD and modulated by FLX and MET [37, 38]. In particular, we focused on IGF2 that is a key molecule in vulnerability to stress and a potential molecular target able to trigger antidepressant action [39–41]. A decrease in IGF2 hippocampal expression is significantly associated to depression-like behavior induced through chronic restraint stress [40–42]. Accordingly, IGF2 overexpression was found to rescue the neurobehavioral effects of stress exposure [40]. In addition, recent evidence indicates that IGF2 administration enhances adult neurogenesis in the hippocampal dentate gyrus [43], considered a marker of recovery from MDD [44, 45], indicating IGF2 as involved in switching from depressive-like to healthy phenotype. IGF2 has been also reported to be a key target of ketamine [39], a novel antidepressant drug, which has a rapid but not long-lasting action [46], similarly to the effect of the combined FLX-MET treatment reported here. This suggests that IGF2 might be involved mainly in the first-phase recovery from MDD. Here, we found that IGF2 is significantly increased by the combined treatment compared to VEH and FLX alone, suggesting that this growth factor might be involved in the antidepressant action of the FLX-MET treatment. However, since IGF2 expression has been associated to enhanced learning and memory [47], the differences in wanting-type anhedonia, assessed through a progressive ratio learning paradigm, could be ascribed also to the differences in learning abilities associated to IGF2 levels. The differences in the IGF2 expression levels concern mainly the dorsal hippocampus. Despite that the classic view on anatomical segregation of the hippocampal function considers the dorsal part to be involved in learning and memory while the ventral part in emotional and stress responses [48, 49], an increasing number of studies are challenging this dichotomy view [50, 51]. Indeed, novel evidence indicates that the dorsal hippocampus is implicated in MDD [52–54] and is an important target for antidepressants [29, 30, 53–55]. For instance, though the ventral region shows the highest expression levels of most markers of antidepressant action, such as the 5HT1A receptor in the dentate gyrus [56], the dorsal region expresses at high levels specific markers, including the 5-HT6 receptor, emerging as relevant regulators of depression-like behavior as well [57, 58].

We also analyzed the expression levels of other metabolic markers related to MDD such as IGF1, p11, BDNF, and leptin, but these were not affected by treatments. Nevertheless, all of them showed a significantly different expression in the two hippocampal regions. The adipose-derived hormone leptin is well known for its function in controlling energy homeostasis and has been recently involved in regulating mood and emotion [59, 60]. Low levels of leptin are associated to depression in humans, and preclinical models as well as pharmacological studies indicate leptin as a potential antidepressant drug [61]. Here, we observe an higher leptin expression in the ventral compared to the dorsal hippocampus. Such specificity is in line with previous data showing that leptin differently affects memory and food intake when administered in the dorsal or the ventral hippocampus [62]. Similar to leptin, the expression of p11 (also known as S100A10), involved in the regulation of depression-like behavior and response to antidepressants [63–65], was not modified by treatments but its expression levels were higher in the ventral compared to the dorsal hippocampus.

BDNF is a neurotrophic factor particularly abundant in hippocampal neurons [66] that has been indicated as a key player in the pathophysiology of MDD. Indeed, according to the “neurotrophic hypothesis of depression,” the psychopathology is associated with the reduction of brain BDNF levels and antidepressant treatments alleviate depressive symptoms increasing its levels [6, 67]. BDNF is reported to be expressed at higher levels in the dorsal compared to the ventral hippocampus [68]. We here confirm this finding. In addition, we replicate data from our and other research groups showing that FLX treatment does not increase the levels of this neurotrophin in a stressful environment [4]. In line with previous data, BDNF levels were not affected also by MET [69]. Akin to IGF1, BDNF levels were higher in the dorsal compared to the ventral hippocampus.

Similar to gene expression, physiological properties differ along the longitudinal axis of the hippocampus. For instance, in the CA1 region [70–72], the LTP magnitude is smaller in the ventral than in the dorsal hippocampus [73–75] and is differentially modulated by stress in the two regions [76–81], being reduced or not affected in the dorsal but increased in the ventral hippocampus following both acute stress [82, 83] and chronic stress [27]. In line with these findings, we show here that the LTP magnitude was smaller in dorsal compared to ventral hippocampus. This might be due to the different distribution and effects exerted by the corticosteroid receptors, mineralocorticoids (MRs) and glucocorticoids (GRs), on LTP after exposure to stress. In particular, it has been reported that MRs, more expressed in the ventral part [84], facilitate LTP [85, 86], while GRs, more abundant in the dorsal part of the hippocampus [84], impair LTP [87].

Interestingly, the treatments differentially affected LTP in the two hippocampal regions. In particular, the FLX-MET cotreatment produced a significant increase in LTP amplitude in the dorsal hippocampus, which parallels the significant IGF2 expression increase observed in this region. Given the role of IGF2 in modulating biological processes involved in neuronal plasticity [88], such as promoting dendritic spine formation [89] and enhancing pERK1/2 and GluR1 [47], the IGF2 increase might be involved in the plasticity enhancement that we observed. By contrast, no significant difference in LTP was found in the ventral part, suggesting that FLX and MET, alone or in combination, do not regulate plasticity in this area. This evidence suggests that the FLX and MET combination affects the electrophysiological activity specifically in the dorsal hippocampus which has been reported as a potential target for antidepressant treatments [29, 30, 53–55]. Such LTP amplitude enhancement, in addition to the increased IGF2 expression, in a brain region reportedly involved in learning processes further supports that these changes might contribute to the differences in the progressive ratio paradigm used to assess wanting-type anhedonia.

The major limitations of the present study include the lack of the analysis of the molecular and cellular endpoints after 1 week of treatment in order to better investigate the association between behavioral changes and modifications in neurophysiological substrates. In addition, a pharmacokinetic analysis of the possible interaction between metformin and fluoxetine would have better illustrated whether the coadministration affects their bioavailability. Finally, given that MDD affects mostly female with a female : male ratio of approximately 2 : 1, the assessment of the effect of the treatments not only in males but also in female individuals will be extremely relevant.

## 5. Conclusions

In conclusion, previous works by us and others have found that FLX administration has beneficial effects in an enriched environment but has no effects or even leads to detrimental outcome when administered in a stressful environment [4, 5, 90]. Here, we show that the combined FLX and MET treatment is more effective than FLX and VEH alone in a short term when administered in individuals exposed to a stressful condition. Therefore, this polypharmacological strategy appears effective to counteract the potential limited efficacy of FLX in individuals living in adverse conditions. This might be highly relevant in the clinic because, with very few exceptions, people cannot rapidly and effectively change their life circumstance and adverse conditions are very common in depressed patients.

## Data Availability

The data used to support the findings of this study are available from the corresponding author upon request.

## Supplementary Materials

Supplementary Materials Figure S1: experimental design of the stressful condition. Mice were exposed to stress for six weeks. The first two weeks induced the depression-like phenotype. During the following four weeks of stress, the subjects were treated with fluoxetine, metformin, or their combination. Figure S2: experimental design and effects of fluoxetine, metformin, or their combination on depression-like behavior in standard condition: (a) first, mice were exposed for two weeks to the stressful condition to induce a depression-like phenotype. Afterwards, they were treated for four weeks in the standard condition. (b) Liking-type anhedonia. Saccharin preference significantly decreased following exposure to stress. Treatments did not produce different effects. (c) Wanting-type anhedonia. The breakpoint level was significantly reduced after the unpredictable chronic mild stress. Treatments did not produce different effects.
