# Supplementary material for: Combined Fluoxetine and Metformin Treatment Potentiates Antidepressant Efficacy Increasing IGF2 Expression in the Dorsal Hippocampus
Source: Neural Plast. 2019 Jan 21;2019:4651031. doi: 10.1155/2019/4651031 (PMC6360645; doi:10.1155/2019/4651031)
Supplement: Supplementary Materials — Figure S1: experimental design of the stressful condition. Mice were exposed to stress for six weeks. The first two weeks induced the depression-like phenotype. During the following four weeks of stress, the subjects were treated with fluoxetine, metformin, or their combination. Figure S2: experimental design and effects of fluoxetine, metformin, or their combination on depression-like behavior in standard condition: (a) first, mice were exposed for two weeks to the stressful condition to induce a depression-like phenotype. Afterwards, they were treated for four weeks in the standard condition. (b) Liking-type anhedonia. Saccharin preference significantly decreased following exposure to stress. Treatments did not produce different effects. (c) Wanting-type anhedonia. The breakpoint level was significantly reduced after the unpredictable chronic mild stress. Treatments did not produce different effects. [file 4651031.f1.pdf]

SUPPLEMENTARY MATERIALS – Poggini et al.

FIGURE S1

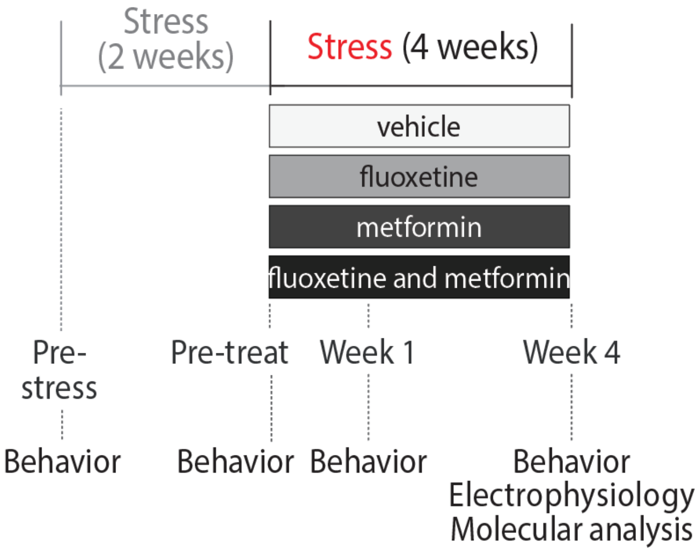

Experimental design of the stressful condition. Mice were exposed to stress for six weeks. The first two induced the depression-like phenotype. During the following four weeks of stress, the subjects were treated for with fluoxetine, metformin or their combination.

FIGURE S2

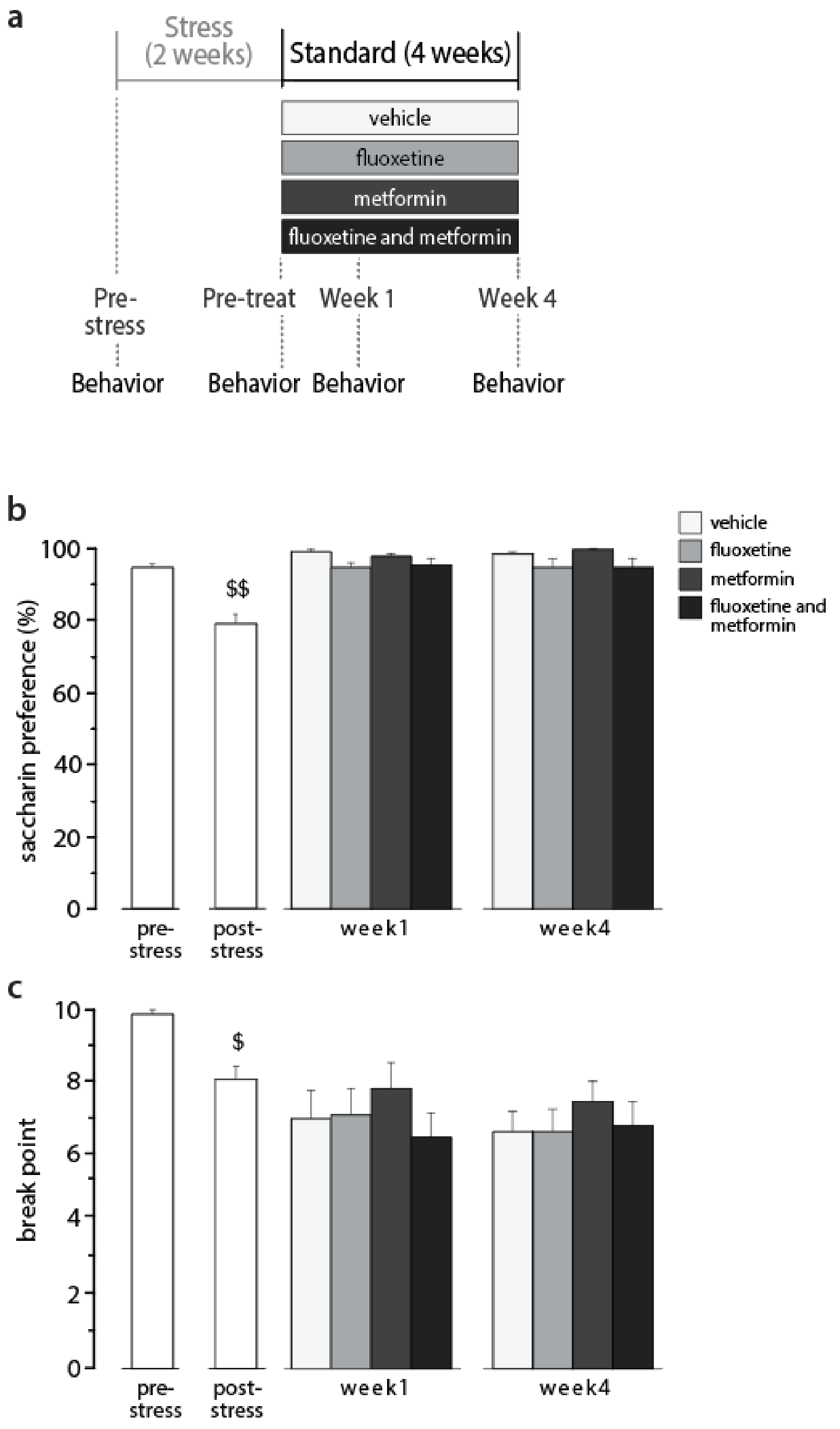

Experimental design and effects of fluoxetine, metformin or their combination on depression-like behavior in standard condition: (a) First mice were exposed for two weeks to the stressful condition to induce a depression-like phenotype. Afterwards, they were treated for four weeks in the standard condition. (b) Liking-type anhedonia. Saccharin preference significantly decreased following exposure to stress. Treatments did not produce different effects. (c) Wanting-type anhedonia. The breakpoint level was significantly reduced after the unpredictable chronic mild stress. Treatments did not produce different effects. Treatments as indicated in the legend, n=9-10 mice per group. $$p<0.0001 and $p=0.0022 pre- vs post-stress. Data are presented as mean + S.E.M.
